# Management of sexual health issues in adolescents and young adults with congenital lower urinary tract malformations: concerns emerging from a survey of Italian pediatric urology centers

**DOI:** 10.3389/fped.2026.1900529

**Published:** 2026-08-25

**Authors:** Rachele Borgogni, Claudia Gatti, Francesca Caravaggi, Ilaria Manghi, Alfredo Berrettini, Dario Guido Minoli, Maria Luisa Capitanucci, Ermelinda Mele, Paolo Caione, Giuseppe Cretì, Evi Comploj, Marcello Carlucci, Massimo Catti, Simona Gerocarni Nappo, Alberto Mantovani, Maria Taverna, Lorenzo Masieri, Giuseppe Masnata, Arianna Lesma, Alessandro Morlacco, Paola Spina, Maria-Grazia Scarpa, Giovanni Torino, Pier Luca Ceccarelli, Vincenzo Domenichelli, Giovanni Mosiello

**Affiliations:** 1 Pediatric Surgery Unit, Arcispedale Sant’Anna, Ferrara, Italy; 2 Pediatric Urology Unit, Azienda Ospedaliero Universitaria Parma, Parma, Italy; 3 Pediatric Surgery Unit, IRCCS Policlinico S. Orsola Bologna, Bologna, Italy; 4 Pediatric Urology Unit, Fondazione IRCCS Ca Granda, Ospedale Maggiore Policlinico, Milano, Italy; 5 Division of Neuro-Urology, IRCCS Ospedale Pediatrico Bambino Gesù, Roma, Italy; 6 Pediatric Urology Unit, IRCCS Ospedale Pediatrico Bambino Gesù, Roma, Italy; 7 Pediatric Urology Unit, Salvator Mundi International Hospital, Roma, Italy; 8 Pediatric Urology Unit, Casa Sollievo Della Sofferenza, San Giovanni Rotondo, Italy; 9 Department of Urology, Central Hospital of Bolzano, Bolzano, Italy; 10 Pediatric Surgery Unit, IRCCS Ospedale Pediatrico Giannina Gaslini, Genova, Italy; 11 Division of Pediatric Urology, Department of Pediatrics and Pediatric Specialties, Regina Margherita Children’s Hospital, Torino, Italy; 12 Pediatric Urology Department, Meyer Children’s Hospital IRCCS, Firenze, Italy; 13 Unit of Urology and Renal Transplantation, Azienda Ospedaliero-Universitaria Careggi, Firenze, Italy; 14 Pediatric Urology and Urodynamics, Arnas G.Brotzu Hospital, Cagliari, Italy; 15 Unit of Pediatric Surgery, Department of Urology, IRCCS Ospedale San Raffaele, Milano, Italy; 16 Urology Clinic, Department of Surgery, Oncology and Gastroenterology, Azienda Ospedaliera Universitaria Di Padova, Padova, Italy; 17 Pediatric Surgery Unit, Azienda Ospedaliero Universitaria Maggiore Della Carita’, Novara, Italy; 18 Pediatric Urology Unit, IRCCS Burlo Garofalo Materno Infantile, Trieste, Italy; 19 Pediatric Urology Unit, Santobono-Pausilipon Children Hospital, Napoli, Italy; 20 Pediatric Surgery Unit, Azienda Ospedaliero Universitaria Di Modena, Modena, Italy; 21 Pediatric Surgery Unit, Ospedale Infermi, Rimini, Italy

**Keywords:** bladder exstrophy, cloaca, fertility, low urinary tract dysfunction, sexuality, spina bifida

## Abstract

**Introduction:**

Transitional care of patients with complex uro-genital malformations (CUGM), including spina bifida, bladder exstrophy-epispadias complex, cloaca, congenital adrenal hyperplasia, and vaginal atresia, remains a worldwide challenge. These conditions are associated with lower urinary tract dysfunction (LUTD), long-term urinary morbidity, and significant psychosocial consequences. The aim of this study was to investigate how pediatric urologists in Italy address sexuality and fertility in adolescents and young adults with CUGM.

**Materials and methods:**

A 23-item multiple-choice-questionnaire was developed by the Italian Society of Pediatric Urology (SIUP) to investigate health care current provider practices regarding sexual health, fertility, gender identity, and transitional care in adolescents and adults with congenital urogenital malformations (CUGM). Each participating center completed one questionnaire after reviewing its actively followed patient cohort. Data were analyzed at the center level using descriptive statistics.

**Results:**

With a 95% response rate, data from 725 patients were collected. Most centers (43%) followed 20–50 patients; 12.5% managed 50–100, and only two followed >100. Multidisciplinary teams were present in 75% of centers, including psychologists (62.5%) and sexologists (25%). All responding centers reported to have in follow-up sexually active patients; 7% of centers reported caring for patients with history of sexual abuse. Sexual health topics were routinely addressed: erectile dysfunction (93%), ejaculation (87.5%), vaginal lubrication (81%), contraception (62.5%), STDs (75%), and pregnancy (75%). Validated questionnaires were applied only in 19% of centers in males and 7% in females. Sexual issues were discussed in the presence of relatives in 31% of centers. 69% of centers reported addressing or identifying substance abuse among their patients. Pelvic organ prolapse screening was performed in 19%. 56% of centers reported following patients identifying as homosexual or bisexual, while transgender identity was reported in 12.5%. Although 69% of centers felt confident discussing gender dysphoria, only 31% were familiar with the GIDYQ-AA; all healthcare professionals expressed the need for additional training on gender identity.

**Conclusion:**

This national provider survey highlights important gaps in the organization of transitional sexual healthcare for patients with CUGM. The findings reflect current practices among Italian pediatric urology centers and support the development of standardized multidisciplinary transition pathways.

## Introduction

Adolescents and young adults affected by severe congenital conditions—such as spina bifida (SB), bladder exstrophy-epispadias complex (BEEC), cloacal anomalies (ARMC), congenital adrenal hyperplasia (CAH), and vaginal atresia (VA)—are now reaching adulthood with an increasing demand for optimal quality of life. Advances in pediatric urology have significantly improved survival, renal preservation, and management of lower urinary tract dysfunction (LUTD), allowing a growing number of patients with complex urogenital malformations (CUGM) to reach adulthood. Nevertheless, many continue to experience urinary incontinence, bladder dysfunction, bowel disorders, repeated surgical interventions, and psychosocial challenges that extend beyond traditional urological outcomes. As this population ages, pelvic floor dysfunction, sexual health concerns, fertility issues, and gender-related questions emerge as increasingly relevant determinants of long-term wellbeing. Urinary incontinence and sexual dysfunction have a particularly detrimental impact on quality of life. Impaired body image, low self-esteem, continence issues, dependence on parental support, and other psychosocial factors may hinder normal psychosexual development throughout childhood and adolescence (1, 2).

In recent years, transitional urology has gained growing attention. A structured transition process aligns with the patient-centred approach promoted by the 24 European Reference Networks (ERNs) for rare diseases established by the European Commission in 2017. As increasing evidence highlights the lifelong impact of LUTD and congenital urogenital anomalies on psychosocial wellbeing, sexuality, and reproductive health, the need for integrated multidisciplinary transitional care has become increasingly evident. Nonetheless, the transition from pediatric to adult care remains a global challenge for both patients and healthcare providers. In many cases, pediatric urologists continue to follow CUGM patients into adulthood within pediatric settings, often due to difficulties in transferring care, particularly regarding sexuality and reproductive health (3).

Despite the recognized importance of these outcomes, little is known about how pediatric urologists address sexuality, fertility, and gender identity during the long-term follow-up of patients with CUGM and associated lower urinary tract dysfunction. To gain insight into current clinical practice, the Italian Society of Pediatric Urology (SIUP) developed a multiple-choice questionnaire (MPCQ) focusing specifically on sexuality, fertility, and gender identity. The aim of this study was to investigate current health care provider-reported practices regarding sexual health, fertility, gender identity, and transitional care in adolescents and adults with CUGM across Italian paediatric urology centres.

## Materials and methods

The final questionnaire investigated the approach of healthcare providers toward sexuality and transitional care in patients with CUGM.

The survey explored the continuity of care from adolescence into adulthood, the presence of multidisciplinary teams and dedicated outpatient clinics, and the involvement of psychologists and sexologists. It also assessed the frequency and context in which sexual health issues were discussed during follow-up visits, including topics such as sexual activity, contraception, sexually transmitted infections, sexual abuse, and substance use. Additional items addressed the management of male and female sexual function, prescription practices for phosphodiesterase type 5 inhibitors, screening for pelvic organ prolapse (POP), occurrence of patients diagnosed with POP, the potential need for surgical management of POP and counselling on bowel management before sexual activity.

The need of a genitoplasty revision carried out with gynaecologists or adult urologists was also investigated.

The use of validated instruments for the assessment of sexual function (IIEF-15, FSFI, FSDS) was recorded.

Finally, questions explored the experience of providers in managing homosexual, bisexual, and transgender patients, their familiarity with gender dysphoria and related assessment tools (GIDYQ-AA), the availability of psychological support, and the perceived need for further training in this field (Table 1).

**Table 1:** 23 items MCQs questionnaire.

Questions	Answers
1) Do you continue to follow CUGM patients through adolescence?	YES <20 pts 20–50 pts 50–100 pts >100	NO
2) Do you follow CUGM patients into adulthood (18 years and above)?	YES maximum age	NO
3) Do you have a multidisciplinary team? A dedicated outpatient’s clinic? Do you have a psychologist in your team? Does your hospital team include a sexologist?	YES YES YES YES	NO NO NO NO
4) Do you have patients who are sexually active?	YES	NO
5) Have any female CUGM patients delivered? If yes, was delivery managed at a tertiary care centre?	YES YES	NO NO
6) During follow-up visits, do you discuss sexual health issues with adolescent/adult CUGM patients? If yes, is the discussion held with: Patients only? Patients and their parents?	YES YES YES	NO NO NO
7) During visits, do you address the following topics? Masturbation First sexual intercourse Sexual abuse Contraception Sexually transmitted infections (STIs) Substance abuse If yes, are patients generally satisfied after these discussions?	YES YES YES YES YES YES YES	NO NO NO NO NO NO NO
8) For male patients: Do you discuss erectile function? Do you discuss ejaculation problems? If yes, do you co-manage these issues with an adult urologist?	YES YES YES	NO NO NO
9) For female patients: Do you discuss vaginal lubrication? Do you address dyspareunia?	YES YES	NO NO
10) Do you feel you need additional training to manage these patients?	YES	NO
11) Do you use validated sexual dysfunction questionnaires for male patients? If yes, which ones (e.g., IIEF-15)?	YES	NO
12) Do you use validated sexual dysfunction questionnaires for female patients? If yes, which ones (e.g., FSFI, FSDS)?	YES	NO
13) Regarding male patients with spina bifida (SB) and bladder exstrophy-epispadias complex (BEEC), are sildenafil or similar drugs prescribed? If yes, are these prescribed by pediatric or adult urologists?	YES	NO
14) Do you routinely screen for pelvic organ prolapse (POP) in female SB and BEEC patients?	YES	NO
15) Do you have patients diagnosed with POP? Have these patients undergone surgical treatment?	YES YES	NO NO
16) Do you provide education on proper bowel management prior to sexual intercourse?	YES	NO
17) In adult patients, is revision genitoplasty performed: Exclusively by your team? In collaboration with gynaecologists/adult urologists?	YES YES	NO NO
18) Do you follow homosexual/bisexual CUGM patients?	YES	NO
19) Are you knowledgeable about gender dysphoria?	YES	NO
20) Are you familiar with the GIDYQ-AA questionnaire?	YES	NO
21) Do you follow transgender patients?	YES	NO
22) Is there a psychologist dedicated to patients with gender dysphoria in your hospital?	YES	NO
23) Do you believe gender identity issues require more in-depth assessment?	YES	NO

A 23-item multiple-choice questionnaire (MCQ) was developed by a multidisciplinary group of clinicians within the SIUP, including pediatric urologists, adult urologists, neuro-urologists, paediatricians, and pediatric surgeons. The questionnaire was based on a review of the existing literature on sexuality and transitional care in patients with CUGM.

The content validity of the questionnaire was assessed using the Content Validity Index (CVI) method. A panel of six experts within the group of the SIUP was invited to evaluate the relevance and clarity of each item.

Each expert rated every question on a 4-point Likert scale (1 = not relevant, 2 = somewhat relevant, 3 = quite relevant, 4 = highly relevant). For each item, the Item-level Content Validity Index (I-CVI) was calculated as the proportion of experts who rated the item as 3 or 4. The Scale-level Content Validity Index (S-CVI/Ave) was obtained by averaging all I-CVI values.

Following Lynn's criteria, items with an I-CVI ≥ 0.78 were considered acceptable. Minor revisions were made to wording and structure based on experts' qualitative feedback before final distribution of the questionnaire. The questionnaire was administered via e-mail to all Pediatric Urology and Pediatric Surgery Unit throughout Italy, encompassing both National Health Service and private institutions. Adults Italian Urology Centres were not included.

Each participating centre completed a single questionnaire summarizing a) number of patients in follow-up; b) its daily routine clinical practice and the characteristics of its actively followed cohort of adolescent and adult patients with CUGM. Healthcare providers reviewed their local clinical records before completing the survey. In order to facilitate IRB approval, only aggregated centre-level information was reported to the coordinating investigators; no identifiable or individual patient-level data were collected or transferred. Consequently, except for the total number of patients followed across participating centres, all analyses were performed at the centre level, and percentages reported in this manuscript refer to the proportion of responding centres unless otherwise specified.

This survey received exemption approval from the Institutional Review Board of a tertiary pediatric hospital (RAP-2025_0003). The study consisted of an anonymous provider survey reporting aggregated centre-level information only. No identifiable patient-level data were collected or transferred to the coordinating investigators. Written informed consent had been obtained by each participating centre according to local institutional policies before inclusion of patients in clinical databases used for the survey.

Descriptive statistics were used to summarize the survey results. Categorical variables are presented as frequencies and percentages. Unless otherwise specified, percentages refer to the proportion of responding centres providing affirmative responses to each questionnaire item.

## Results

The average proportion of items judged as relevant across the six experts was 0.85 with a S-CVI/Ave equal to 0.91.

The questionnaire achieved a 95% response rate, 5% of Centres decided not to participate.

Unless otherwise specified, the percentages reported below refer to responding centres rather than individual patients. In 93% of centres, patients older than 18 years were followed.

Participating centres collectively reported following 725 patients aged 16–40 years at the time of the survey.

All centres managed the full spectrum of CUGM conditions (SB, BEEC, CAH, cloaca, and VA), except one, which treated only BEEC. Five centres (31%) followed fewer than 20 patients; 43% managed 20–50, 12.5% followed 50–100, and two centres (12.5%) cared for over 100 patients.

The period over which the data were collected was 6 months.

A multidisciplinary team was available in 75% of centres, and 62.5% had a dedicated outpatient clinic. A psychologist was part of the team in 62.5% of cases, while a sexologist was included in 25%.

Sexuality was addressed by 93% of healthcare providers (HCPs). Interviews were conducted with both patients and parents in 62% of cases; only 7% of centres interviewed patients alone.

87.5% of healthcare providers considered their patients generally satisfied after these discussions.

Sexual dysfunction was routinely investigated: 93% of centres routinely discussed erectile dysfunction during follow-up, with 25% of centres managed in collaboration with adult urologists. In females, vaginal lubrication and dyspareunia were addressed in 81% and 54% of cases. Validated tools were applied in a minority: the IIEF-15 in 19% of males and FSFI in 7% of females. Phosphodiesterase type 5 inhibitors (e.g., sildenafil) were prescribed in 25% of participating centres; half were prescribed by adult urologists.

POP screening was performed only by 19% of centres. 27% of centres reported following patients diagnosed with POP. Bowel management before sexual intercourse was routinely discussed in 87.5% of centres. Genitoplasty revisions in adulthood were managed in collaboration with gynaecologists or adult urologists in only 25% of cases, in the remaining cases, due to the absence of a structured transitional care pathway, the patients continue to be followed by the pediatric team.

56% of centres reported following patients identifying as homosexual or bisexual, and Transgender identity was investigated and reported in 12.5% of centres. Gender dysphoria was considered by 69% of HCPs, though only 31% were familiar with the GIDYQ-AA questionnaire. All HCPs agreed on the need for greater focus and training on gender identity-related care (Table 2).

**Table 2:** Centre-level responses to the survey.

Topics analyzed	YES	NO
Sexually active	100.0%	0.0%
Masturbation	56.0%	44.0%
First intercourse	81.0%	19.0%
Sexual abuses	7.0%	93.0%
Contraception	62.5%	37.5%
Knowledge of sexually transmitted diseases	75.0%	25.0%
Substance abuse	69.0%	31.0%
Pregnancy	75.0%	25.0%
Pregnancy in tertiary center	100.0%	0.0%

## Discussion

Historically, the primary aim in managing patients with complex uro-genital malformations (CUGM) was to reduce morbidity and mortality while preserving renal function and achieving urinary continence. Advances in reconstructive surgery, bladder management, and the treatment of lower urinary tract dysfunction (LUTD) have dramatically improved survival and long-term outcomes (1, 2). As a consequence, increasing numbers of patients with conditions such as spina bifida, bladder exstrophy-epispadias complex, cloacal malformations, and other congenital urogenital anomalies are now reaching adulthood. This epidemiological shift has expanded the focus of care beyond traditional urological outcomes toward broader aspects of quality of life, including sexual function, fertility, body image, intimate relationships, and gender identity. These domains are increasingly recognized as essential components of comprehensive long-term care (3).

Adolescents with CUGM often express a desire for intimate relationships and sexual experiences. However, sexual and reproductive outcomes remain poorly understood and underexplored in this population (4). As highlighted by Bower et al. and more recently by Van den Eede et al., pediatric urologists play a pivotal role in initiating conversations about sexual function, promoting normalization, and offering practical support when difficulties arise (5, 6).

Despite growing awareness, the management of sexuality and fertility in CUGM patients remains a global challenge. In Italy, the national healthcare framework (DM 70/2015) recognizes pediatric urology as a distinct subspecialty, organizationally separate from both pediatric surgery and adult urology (7). However, discrepancies in the legal definition of pediatric age across Italian regions (ranging from 14 to 18 years) hinder standardized, lifelong multidisciplinary care. Furthermore, many pediatric urology units are housed in standalone pediatric hospitals, often lacking integration with adult services—complicating the transition process.

Our survey suggested many issues useful for improving care and cure on long-term follow-up of patients with CUGM:

1. Organization of National Health System.

A high number of adult CUGM patients continue to receive follow-up care in pediatric settings. This was the premise for conducting our multicentre investigation, aimed at assessing how sexual health is evaluated and managed in this cohort. While our findings are limited by the study design, they nonetheless provide valuable insights into provider practices and perceptions.

2. Multidisciplinary team and its composition.

A positive finding is the widespread presence of multidisciplinary teams across responding centres, with psychologists frequently involved. However, sexologists remain underrepresented. This finding is particularly relevant because sexual health outcomes in CUGM patients are closely intertwined with continence status, body image, self-esteem, and social participation. Previous studies in patients with congenital lower urinary tract dysfunction have demonstrated that urinary incontinence and dependence on bladder management strategies may negatively affect intimate relationships, psychosocial adjustment, and sexual wellbeing. Despite this recognized interplay, dedicated psychosexual support remains inconsistently available during follow-up and transition programs.

3. Outpatient clinic organization.

Moreover, discussions of sexual health are often conducted in the presence of parents or multiple healthcare professionals, which may limit patient openness and confidentiality. This dynamic reflects findings from other studies highlighting the strong role of families in clinical interactions (8–10). Healthcare professionals should therefore strive to improve patient-provider communication and preserve adolescent privacy in sensitive discussions.

4. Gender difference.

Notably, sexual dysfunction is investigated more systematically in male than in female patients. While issues such as contraception and sexually transmitted infections (STIs) are commonly addressed, limited attention is devoted to topics like sexual or substance abuse, despite their close association with psychological well-being (11). The importance of structured psychosexual support in promoting emotional and sexual development has been highlighted by several studies conducted in patients with complex uro-genital malformations (CUGM) (12). In the literature, the majority of research has focused on spina bifida and bladder exstrophy-epispadias complex (BEEC). A recent study by Paraboschi et al. used the validated SESAMO questionnaire to explore individual and couple dynamics in BEEC patients, emphasizing the need to identify psychosexual difficulties to tailor psychological interventions (13). Despite the availability of tools such as the International Index of Erectile Function (IIEF-15), the Female Sexual Function Index (FSFI), and the Female Sexual Distress Scale (FSDS), these remain underutilized in routine follow-up, findings echoed by our data, where fewer than 20% of centres reported using them (14, 15).

Erectile dysfunction is typically evaluated by pediatric urologists; however, treatment, most often with sildenafil, is frequently initiated by adult urologists. It remains unclear from our data whether this referral occurs promptly or with delay.

5. Adult specialist involvement.

Pelvic organ prolapse (POP) is also a relevant concern, particularly in women with SB and BEEC, where it presents earlier, more severely, and in nulliparous patients (16–19). In our survey, 27% of centres reported caring for patients diagnosed with POP, yet only 19% of Centres performed regular screening, highlighting the need for closer collaboration with gynaecologists during transition.

6. Gender identity.

The past decade has seen a notable increase in referrals to gender clinics among adolescents and young adults, reflecting greater visibility and diversity in gender identity (20, 21). Comprehensive sexual health support must therefore include evaluation of sexual orientation and gender identity.

This survey represents the first attempt to investigate gender identity issues in patients with complex uro-genital malformations (CUGM). More than half of responding centres reported following some patients identifying as homosexual or bisexual, suggesting that paediatric urologists should be prepared to address issues related to sexual orientation within transitional care. Despite most healthcare providers acknowledging the relevance of gender dysphoria, familiarity with standardized tools such as the GIDYQ-AA (22) remained limited, highlighting the need for enhanced education and training on gender identity–related care, which is essential for delivering individualized, psychologically supportive care plans.

### Final recommendation

The Italian Society of Pediatric Urology, based on these results, suggests that sexual health, fertility counselling, and gender identity should be considered integral components of transitional care pathways for patients with CUGM. While preservation of renal function and management of lower urinary tract dysfunction remain fundamental objectives, these outcomes alone do not fully capture patients' long-term wellbeing. As survival and continence outcomes continue to improve, healthcare providers must increasingly address psychosexual development, reproductive health, and gender-related concerns through structured multidisciplinary programs. Greater collaboration among pediatric and adult urologists, gynaecologists, psychologists, sexologists, and other allied professionals may facilitate a more comprehensive and patient-centred model of care.

National Health System organization should consider improvement of care and cure of fertility and sexuality issues in adolescents and young adults with CUGM, improving program of transitional care, including patient's representatives.

### Limitations

This study has several limitations. First, the questionnaire was administered exclusively to healthcare providers (HCPs), without directly including the perspectives of patients or their families. Although this approach minimized the collection and transfer of patient-level information and protected patient confidentiality, it limited our ability to compare healthcare providers’ perceptions with patients' own experiences and perspectives.

Second, the survey relied on provider-reported information derived from local clinical practice and record review. Consequently, recall bias, variability in clinical documentation, and differences in reporting across participating centres cannot be excluded. Third one, the questionnaire was designed to describe provider practices and centre experience rather than to collect individual patient-level data or estimate the prevalence of clinical or psychosocial characteristics among patients with CUGM. Therefore, the findings should be interpreted as reflecting current clinical practice rather than epidemiological estimates.

Furthermore, patient satisfaction was assessed according to healthcare providers' perceptions rather than through validated patient-reported outcome measures.

Finally, the study was restricted to Italian centres, precluding cross-national comparisons. Given the variability in healthcare organization, socioeconomic factors, and cultural attitudes toward sexuality and transitional care, a universal model may not be applicable. Nevertheless, larger international studies involving both healthcare providers and patients would be valuable to develop standardized, evidence-based transitional care pathways.

## Conclusion

This national survey provides an overview of current provider-reported practices regarding sexual health and transition care in Italian paediatric urology centres. This preliminary national analysis underscores the lack of standardized transitional care pathways addressing sexual health in adolescents and adults with CUGM, a population frequently affected by lifelong lower urinary tract dysfunction (LUTD), continence-related challenges, and complex psychosocial needs, even within specialized pediatric urology centres.

Our findings emphasize the need for a structured, multidisciplinary approach involving sexologists, psychologists, gynaecologists, adult urologists, and other healthcare professionals to ensure comprehensive and continuous care. Tailored protocols that incorporate psychosexual development, gender identity, patient autonomy, and reproductive counselling are essential to facilitate a smooth transition to adulthood and to complement traditional urological goals such as preservation of renal function and management of LUTD.

By identifying existing gaps in current practice, this study provides a foundation for the development of standardized follow-up strategies aimed at preventing psychosexual distress and improving long-term quality of life. Future transitional care pathways should integrate sexual health assessment alongside functional and continence outcomes, promoting a more comprehensive and patient-centred model of care for individuals living with CUGM.

## Data Availability

The raw data supporting the conclusions of this article will be made available by the authors, without undue reservation.
